# Predicting genetic interactions with random walks on biological networks

**DOI:** 10.1186/1471-2105-10-17

**Published:** 2009-01-12

**Authors:** Kyle C Chipman, Ambuj K Singh

**Affiliations:** 1 Biomolecular Science and Engineering Program, UC Santa Barbara, Santa Barbara, CA, USA; 2 Department of Computer Science, UC Santa Barbara, Santa Barbara, CA, USA

## Abstract

**Background:**

Several studies have demonstrated that synthetic lethal genetic interactions between gene mutations provide an indication of functional redundancy between molecular complexes and pathways. These observations help explain the finding that organisms are able to tolerate single gene deletions for a large majority of genes. For example, system-wide gene knockout/knockdown studies in *S. cerevisiae *and *C. elegans *revealed non-viable phenotypes for a mere 18% and 10% of the genome, respectively. It has been postulated that the low percentage of essential genes reflects the extensive amount of genetic buffering that occurs within genomes. Consistent with this hypothesis, systematic double-knockout screens in *S. cerevisiae *and *C. elegans *show that, on average, 0.5% of tested gene pairs are synthetic sick or synthetic lethal. While knowledge of synthetic lethal interactions provides valuable insight into molecular functionality, testing all combinations of gene pairs represents a daunting task for molecular biologists, as the combinatorial nature of these relationships imposes a large experimental burden. Still, the task of mapping pairwise interactions between genes is essential to discovering functional relationships between molecular complexes and pathways, as they form the basis of genetic robustness. Towards the goal of alleviating the experimental workload, computational techniques that accurately predict genetic interactions can potentially aid in targeting the most likely candidate interactions. Building on previous studies that analyzed properties of network topology to predict genetic interactions, we apply random walks on biological networks to accurately predict pairwise genetic interactions. Furthermore, we incorporate all published non-interactions into our algorithm for measuring the topological relatedness between two genes. We apply our method to *S. cerevisiae *and *C. elegans *datasets and, using a decision tree classifier, integrate diverse biological networks and show that our method outperforms established methods.

**Results:**

By applying random walks on biological networks, we were able to predict synthetic lethal interactions at a true positive rate of 95 percent against a false positive rate of 10 percent in *S. cerevisiae*. Similarly, in *C. elegans*, we achieved a true positive rate of 95 against a false positive rate of 7 percent. Furthermore, we demonstrate that the inclusion of non-interacting gene pairs results in a considerable performance improvement.

**Conclusion:**

We presented a method based on random walks that accurately captures aspects of network topology towards the goal of classifying potential genetic interactions as either synthetic lethal or non-interacting. Our method, which is generalizable to all types of biological networks, is likely to perform well with limited information, as estimated by holding out large portions of the synthetic lethal interactions and non-interactions.

## Background

Remarkably, only 18 percent of *S. cerevisiae *genes are known to be essential for viability [1,2], as determined by single gene deletions for nearly all of the predicted 6, 000 genes. Similarly, genome-wide RNAi knockdown experiments conducted in *C. elegans *produced non-viable phenotypes for 10% of the 18, 000 tested genes [3]. The remaining genes that are not required for viability under laboratory conditions are termed "non-essential," though their status more likely reflects the extent to which individual genes can compensate for one another in the event of a null mutation. The concept of genetic buffering [4,5] has received support from recent studies utilizing high-throughput methods (SGA, dSLAM) [6-10] to systematically implement double null mutations for large sets of gene pairs. One major finding of these systematic studies is the prevalence of synthetic sick or lethal (SSL) interactions. SSL interactions are revealed when two genes that are not essential for viability as single loss-of-function mutants combine to form a double mutant with a lethal phenotype.

A key finding in one of the original system-wide studies conducted by Tong *et al*. [7] is that genetic interactions tend to run orthogonal to physical interaction. In light of this observation, several recent studies have sought to model this phenomenon in the context of biological networks [11-13]. Kelley and colleagues [11] used probabilistic models to validate the observation that genetic interactions are often oriented orthogonally to physical interactions, and therefore can be modeled as "between-pathway" interactions. This interpretation is consistent with the theory that genetic buffering confers robustness to molecular complexes and pathways functioning in parallel. The authors also found that, in some cases, genetic interactions may overlap with physical interactions, and can therefore be modeled as "within-pathway" models. This is consistent with an earlier finding that 1 percent of gene pairs exhibiting a SSL interaction also share a physical interaction [7], which is 35 times more frequent than would be expected by chance. Protein complexes enriched for genetic interactions tend to indicate that a particular complex is essential, most likely due to a lack of buffering partners. Finally, Ye and colleagues [12] offer additional evidence supporting the notion that genetic redundancy can be interpreted at the complex level, as they use the congruence of synthetic lethal interactions, defined as the similarity in SSL partners in a genetic interaction network, to predict complex membership. A common theme developed in these studies is that genetic redundancy is to a large extent defined at the level of molecular complex, a property that can be exploited to predict novel interactions.

In addition to the aforementioned studies that used physical interaction data to model synthetic lethal interactions, recent work has demonstrated that synthetic lethal interactions can be leveraged to resolve molecular complexes [8,14-16]. In one study, Collins *et al*. utilized genetic interaction data to provide finer resolution on the molecular function of 743 genes involved in various aspects of *S. cerevisiae *chromosome biology. The authors constructed an epistatic miniarray profile (eMAP) from an exhaustive test of pairwise interactions, from which they were able to characterize the extent to which physically interacting proteins act coherently in a common function. The results from this study suggest that genes that have been systematically tested to interact physically are more likely to form a stable complex if they share common genetic interactions. Similarly, St. Onge *et al*. implemented 650 double deletion experiments corresponding to an exhaustive pairings of 26 genes related to DNA repair. By measuring the fitness of the double deletion strains in the presence of DNA damaging chemicals, the authors were able to detect previously unknown functional relationships and pathway orderings [15]. Thus, these studies collectively suggest that physical interaction data can be used to model genetic interactions, and, conversely, genetic interaction data can be leveraged to provide greater resolution to molecular complexes and pathways that have been inferred from systematic protein-protein interaction and gene co-expression data.

Despite the considerable benefits of high-throughput methods such as SGA and dSLAM, the adoption of SSL screens into the standard toolbox of molecular geneticists would impose considerable cost and time requirements. For example, in order to experimentally map out pairwise gene interactions for the *S. cerevisiae *genome, an exhaustive search would mandate (6, 000 × 6, 000)/2 = 18 million double null experiments. In the case of *C. elegans*, one would need to implement (20, 000 × 20, 000)/2 = 200 million experiments to cover all pairwise interactions. This understates the complexity of such an undertaking, as experimentalists need to account for varying culture conditions and hypomorphic alleles for essential genes. Considering these practical limitations, computational techniques that predict genetic interactions are of potential value in providing molecular biologists with leading candidates for pairwise interactions. Towards this goal, Roth and colleagues [17] reported success using topological information in conjunction with functional genomic information, which was used to build a decision tree-based classification system. Interestingly, it was not the functional genomic data but the 2-hop network characteristics that conferred the strongest predictive power. 2-hop network motifs capture the relationship between a pair of genes, e.g. A-B, and a third gene, C. In this example, genes A and B share a physical interaction, while genes A and C are synthetic lethal. The 2-hop scheme would suggest that genes B and C might also be synthetic lethal. Building on this concept, we apply random walks on biological networks to expand genome coverage and prediction accuracy. Furthermore, we incorporate SSL interactions as well as all experimentally validated non-interactions into our algorithm for measuring topological relatedness, resulting in increased prediction accuracy. Our method is capable of detecting SSL relationships for both the "between-pathway" and "within-pathway" topologies (see "Approach"). We report considerable performance gains in predicting SSL gene interactions as characterized by ROC curves.

## Approach

Our technique is initiated by performing random walks on the individual biological networks, producing proximity matrices for each of the networks. Subsequently, the proximity matrices are combined with the genetic interaction data during the procedure for measuring the topological relatedness between two genes, which is run separately on both the synthetic sick or lethal genetic interaction dataset and the dataset of experimentally tested non-interactions. As a result of this procedure, there are two variables for each biological network (SSL interactions and non-interactions), which are ultimately incorporated into the decision tree classifier as a feature vector to predict genetic interactions.

### Random walks

The random walk procedure with restarts is a computationally efficient method to profile the neighborhood of a node [18]. A biological network, *G*, is represented by *G *= (*V*, *E*), where *V *represents the nodes (genes) and *E *represents the edges (significant linkages between genes). The restart node, *s*, takes on a restart probability, *c *= 0.2, and the procedure is run separately for each node in the biological network. Ultimately, *V *and *E *are translated into a column-normalized proximity matrix, *P*, which is subsequently used to solve for the stationary vector $\vec{p_{s}}$. The stationary vector $\vec{p_{s}}$ represents the steady-state distribution of the neighborhood for a particular node. An overview of the procedure is provided below.

**Input: **The biological network *G *= (*V*, *E*);

a start node *s*;

restart probability *c*;

**Output: **The proximity matrix *P*;

Let $\vec{r_{s}}$(*V*) be the restart vector with value 0 for all of its entries except a 1 for the entry denoted as node *s*;

Let **A **be the column normalized adjacency matrix as defined by the edge matrix, *E*;

Initialize $\vec{p_{s}}$(*V*) = $\vec{r_{s}}$ (*V*);

*Solve for: $\vec{p_{s}}=(1-c)A\vec{p_{s}}(V)+c\vec{r_{s}}(V)$;

*The stationary vector can be obtained by either solving for the dominant eigenvector or running iteratively until convergence. [18].

### Algorithm for measuring topological similarity

**Figure 1:**
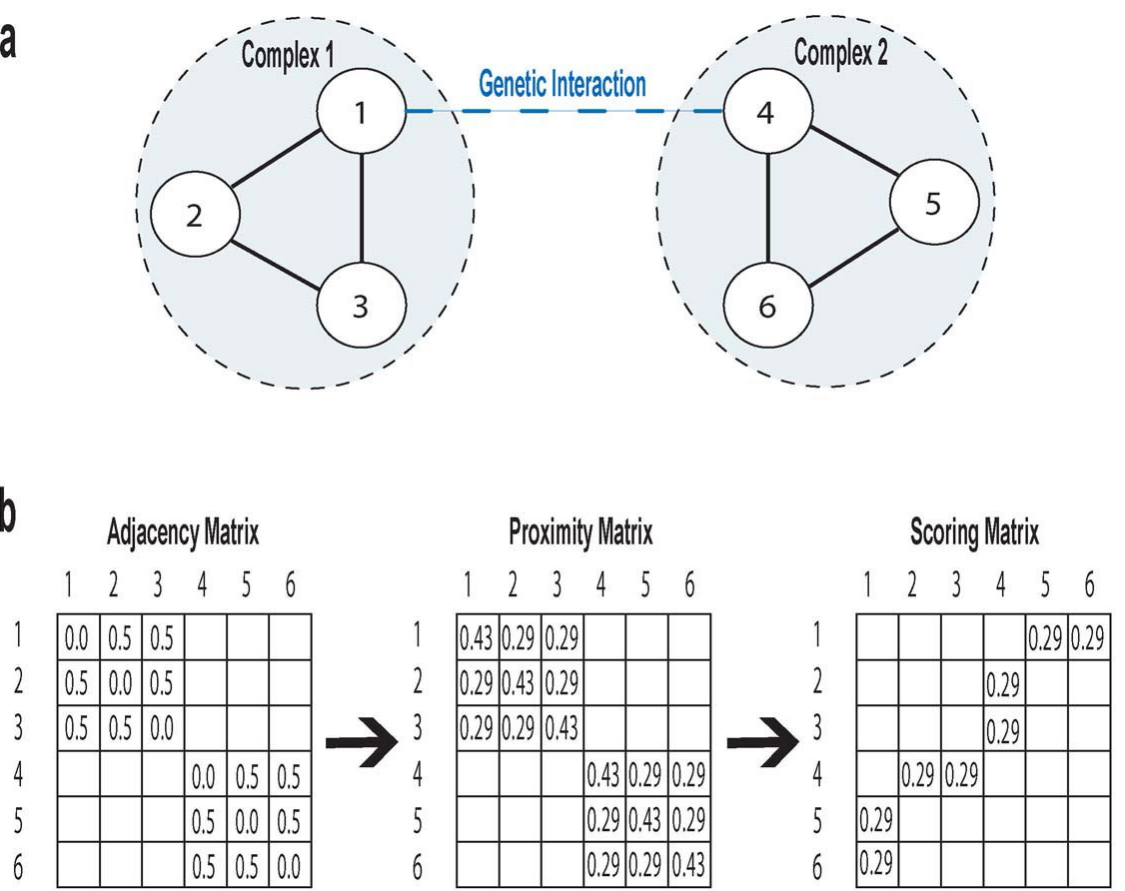
**Methodology**. **a) **Genes 1 and 4 share a genetic interaction that bridges two distinct complexes. Solid lines represent edges in a generalized biological network (PPI, GO, etc.). Dashed lines represent genetic interactions. **b) **An adjacency matrix is derived from the solid lines defining the biological network. We apply our random walk algorithm on the adjacency matrix with a restart probability of 0.2, producing the random walk (proximity) matrix. To generate a scoring matrix (topological relatedness), we iterate through each of the genetic interactions, associating the neighborhood of the interacting gene with its partner.

Performing the random walk procedure produces proximity matrices for each of the biological networks, which are subsequently used to quantify the likelihood of potential genetic interactions. We applied an algorithm to measure the topological relatedness between two genes that iterates through each gene pair in the SSL dataset as well as the non-interaction dataset (pairs that are experimentally determined to not interact). Figure 1 illustrates a simplified case where the genetic interaction dataset consists of one entry of an interaction between genes 1 and 4. Figure 1a portrays a biological network with 6 genes connected by 6 edges, forming two distinct complexes. The genetic interaction between genes 1 and 4 runs orthogonal to the two complexes. Therefore, the neighbors of genes 1, which are genes 2 and 3, will be associated with gene 4. Likewise, genes 5 and 6 will be implicated with gene 1. The strength of the predicted association between genes 2 and 4, for example, will depend on the proximity between genes 1 and 2. As previously mentioned, the proximity between two nodes is determined by the random walk procedure. We find that this system uses the natural information flow inherent to a biological network to predict likely genetic interactions, which is suitable for detecting both intra- and inter-cluster interactions. If, in contrast to figure 1a, a protein complex were significantly enriched for genetic interactions ("intra-complex"), our procedure would implicate genes in the local neighborhood (within the complex). For each of the biological networks, we generate two separate matrices for the interaction and the non-interaction datasets. These procedures are run separately, and the respective measures from the SSL and non-interaction datasets are considered independently by the decision tree classifier in order to optimize the relative weights between the two types of interactions. The data were partitioned 5-fold prior to running the algorithm, such that the testing instances, comprising 20% of the interactions and non-interactions, were not included in the measurement of topological similarity.

### Classification

We used version 3.5.7 of the Weka [19] machine learning software to classify gene pairs as either interacting (SSL) or non-interacting. Specifically, we used the J48 decision tree implementation provided with the package. We applied a 5-fold, stratified cross-validation scheme whereby four fifths of the instances are used for training and the other one fifth of the data is held out for testing (see above).

### Scoring of gene pairs

Each gene pair is assigned a probability according to the leaf to which it is directed. Each leaf in the decision tree is associated with a probability according to the ratio of interacting pairs versus the total number of gene pairs assigned to that leaf during the training process. In order to generate ROC curves, we varied the threshold probability associated with the "SSL/interacting" class by a factor of 0.0001 over a range of 0 to 1, thereby generating 10, 000 data points for each ROC curve.

## Results and discussion

We compared the performance of our random walk-based method to the leading methods of Wong *et al*. [17] and Zhong *et al*. [20]. We note that while other existing studies have successfully modeled genetic interactions [11,13], these techniques are not optimized for predicting novel interactions and are therefore not incorporated into our performance measurements. We first offer a comparison of the random walk method against the two established methods. Subsequently, for the random walk method, we show the predictive ability of each of the individual datasets in both *S. cerevisiae *and *C. elegans*, the added value provided by non-interaction data, and the robustness of our method under conditions where varying levels of information are held out. The performance gain associated with our method is present in both *S. cerevisiae *and *C. elegans*.

### Comparison to established methods

**Figure 2:**
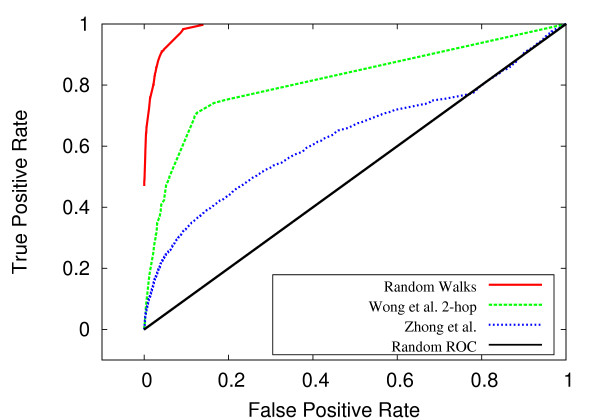
**ROC comparison for *C. elegans***. For *C. elegans*, ROC curves for the random walk, 2-hop (Wong *et al*.), and Zhong *et al*. methods.

**Figure 3:**
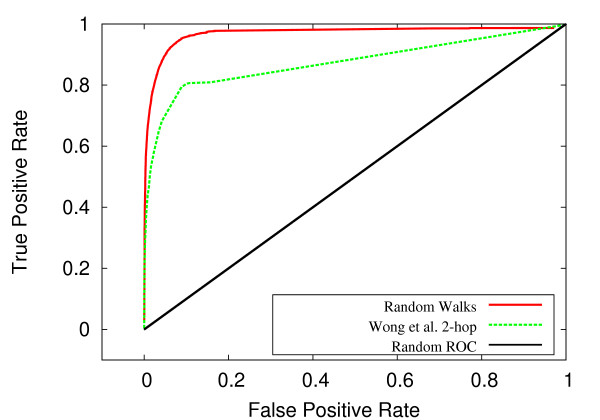
**ROC comparison for *S. cerevisiae***. For *S. cerevisiae*, ROC curves for the random walk and 2-hop (Wong *et al*.) methods.

Using standard receiver operating characteristic (ROC) curves, our random walk method was compared to the 2-hop method of Wong *et al*. [17] and to the more recent method of Zhong *et al*. [20]. For both the *S. cerevisiae *and *C. elegans *systems, the random walk method outperformed these two methods by a considerable margin. For *S. cerevisiae*, we achieved an area under ROC curve (AUC) of .969 versus .874 for the 2-hop method. For *C. elegans*, we achieved 0.984 with our method, compared to 0.793 for the 2-hop technique and 0.630 for the Zhong *et al*. approach. Figures 2 and 3 show the ROC curves for *S. cerevisiae *and *C. elegans*, respectively. Expanded network coverage and more accurate measurement of network proximity are the most likely explanations for the performance improvement over the 2-hop approach. The lower performance of the Zhong *et al*. method most likely reflects the fact that this method is geared towards predicting close-range functional relationships, due to reliance on likelihood scoring of isolated gene pairs. Stated differently, to glean insight into the possibility of a genetic interaction between genes A and B, the Zhong *et al*. method requires prior evidence of an interaction (e.g. co-expression, GO annotation) between genes A and B. At the same time, prior evidence regarding genes A and B can only be used to implicate genes A and B. In contrast, the random walk method considers, for each data type, the network neighborhood of individual genes in an interacting pair, thus allowing the cross-pollination of information between genetic interaction data and other biological data represented as networks.

### Integrating the biological networks

**Figure 4:**
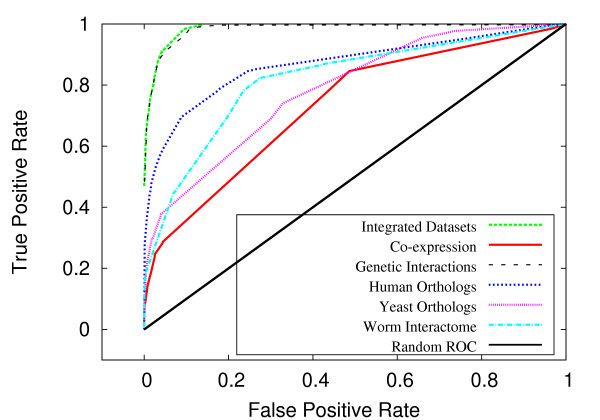
**ROC performance breakdown for *C. elegans***. For *C. elegans*, a breakdown by the individual biological datasets.

**Figure 5:**
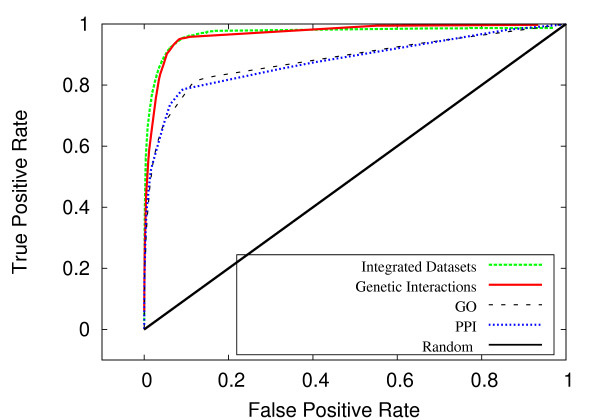
**ROC performance breakdown for *S. cerevisiae***. For *S. cerevisiae*, a breakdown by the individual biological datasets.

Figures 4 and 5 exhibit the classification accuracy for the individual biological networks. In both *S. cerevisiae *and *C. elegans*, the genetic interaction data was the most informative for the purposes of predicting genetic interactions, consistent with the results from Wong *et al*. [17]. In *C. elegans*, worm protein-protein interactions were second to genetic interactions in terms of predictive ability, followed by physical interactions between homologs in human and yeast (figure 4). In yeast, GO interactions and protein-protein interactions were roughly equivalent and were secondary to that of genetic interactions in predictive power (figure 5).

### Non-interaction data improves performance

**Figure 6:**
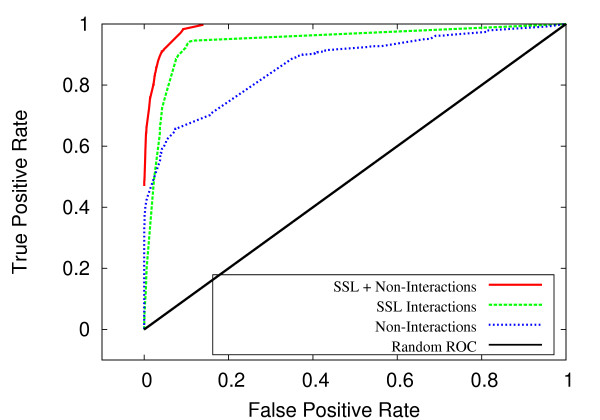
**ROC comparison by SSL and non-interactions for *C. elegans***. For *C. elegans*, ROC curves for SSL interactions, non-interactions and combined SSL + non-interactions.

**Figure 7:**
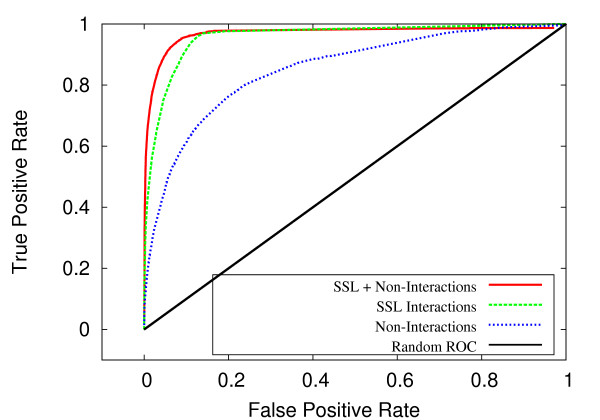
**ROC comparison by SSL and non-interactions for *S. cerevisiae***. For *S. cerevisiae*, ROC curves for SSL interactions, non-interactions and combined SSL + non-interactions.

An important finding of this study is that classification performance is improved by including experimentally validated non-interactions into our algorithm for measuring topological similarity. This value is incorporated into the feature vector as an additional variable. Figures 6 and 7 show that the combination of synthetic sick or lethal (SSL) interactions and non-interactions, when combined, achieve a much greater area under ROC curve. For *C. elegans*, the SSL-only and non-interaction-only data produced AUC values of 0.938 and 0.858, respectively, whereas classification on the combined data resulted in an AUC of 0.984. For *S. cerevisiae*, the SSL-only and non-interaction-only data produced AUC values of 0.952 and 0.866, respectively, whereas the classification on the combined data produced an AUC of 0.969. These results suggest that the density of non-interactions between complexes is indicative of a lack of redundancy, just as SSL interactions are suggestive of redundancy between complexes.

### Performance as a function of available information

In addition to measuring the area under the ROC curves, one may gain insight into the relative power of the respective methods by quantifying classification performance with varying levels of information, where the amount of "information" represents the fraction of interactions and non-interactions that are utilized by the procedure for measuring network relatedness. For example, in the case where 20% of the information is incorporated into the algorithm for measuring topological similarity, 4 out of 5 instances will be included in the algorithm.

**Figure 8:**
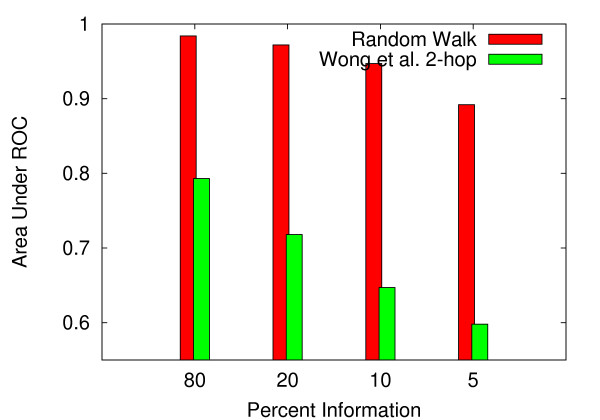
**AUC as a function of information used in the measurment of topological relatedness for *C. elegans***. For *C. elegans*, AUC as a function of information used in the measurment of topological relatedness.

**Figure 9:**
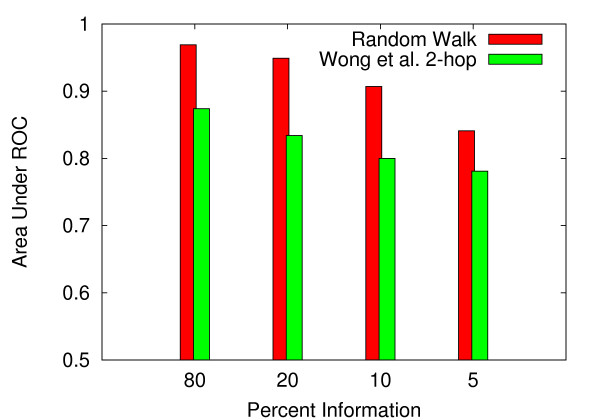
**AUC as a function of information used in the measurment of topological relatedness for *S. cerevisiae***. For *S. cerevisiae*, AUC as a function of information used in the measurment of topological relatedness.

By varying the fraction of interactions and non-interactions utilized by the procedure for measuring network relatedness, we found our system to be fairly robust to markedly reduced information. Figures 8 and 9 indicate that the effects of reducing information from 80 percent to 20 percent are relatively small for both organisms. In the case of *S. cerevisiae*, the area under the ROC curve is reduced from 0.969 to 0.949 upon reducing the information from 80 percent to 20 percent (figure 8). Similarly, in *C. elegans*, the AUC is reduced from 0.984 to 0.972 upon reducing the information from 80 to 20 percent (figure 9). In both organisms, the random walk-based classifier outperforms the 2-hop method across all levels of information. Interestingly, in both *S. cerevisiae *and *C. elegans*, the random walk method utilizing 10 percent of the information outperforms the 2-hop method using 80 percent of the information (figures 8 and 9).

### Controlling for biases in the genetic interaction datasets

**Figure 10:**
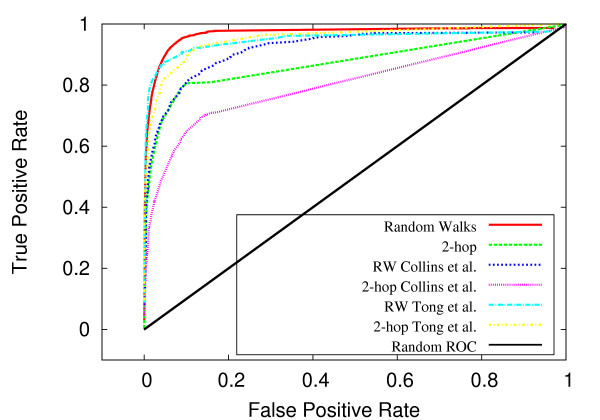
**Comparison of the random walk and 2-hop methods for the composite dataset and Collins and Tong subsets**. For *S. cerevisiae*, ROC curves for the Random Walk and 2-hop techniques. Generally, analysis on the two symmetric subsets produces slightly weaker results as compared to the composite dataset.

**Figure 11:**
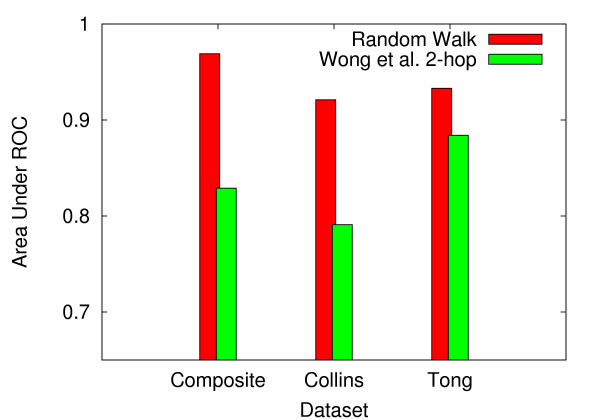
**AUC values for the random walk and 2-hop methods when applied to the composite dataset and the Collins and Tong subsets**. For *S. cerevisiae*, AUC values for the Random Walk and 2-hop techniques. Generally, analysis on the two symmetric subsets produces slightly weaker results as compared to the composite dataset.

To date, the majority of studies conducting tests for synthetic sickness or lethality consist of a small set of query genes crossed against a larger set of target genes. Consequently, the resulting datasets are asymmetric, and it is imperative to consider the possibility that the random walk method might actually be biased towards predicting query-query interactions rather than genetic interactions. To control for this possibility, we ran our analysis on two distinct symmetric subsets of the *S. cerevisiae *composite genetic interaction dataset. First, we utilized the naturally symmetric dataset from Collins *et al*., which consists of 746-by-746 query gene interactions. Next, we derived a 132-by-132 symmetric dataset from the 132 query genes in the Tong *et al*. dataset. Analysis on both of the symmetric subsets produced slightly lower AUC scores as compared to composite dataset. The random walk method, which scored an AUC of 0.969 for the composite dataset, produced AUC values of 0.926 and 0.934 for the Collins *et al*. and Tong *et al*. subsets, respectively (figures 10 and 11). The 2-hop method, which scored an AUC of 0.874 for the composite dataset, produced AUC values of 0.822 and 0.844 for the Collins *et al*. and Tong *et al*. subsets, respectively (figure 10). These results suggest that the asymmetric nature of composite dataset might produce a small degree of bias towards predicting query-query interactions.

## Discussion

To date, the strongest predictor of genetic interactions has invariably been based on information related to network topology combined with knowledge of already established genetic interactions [11,17]. While it would ideal to be able to predict genetic interactions without the knowledge of existing genetic interactions, the predictive ability of methods lacking this information has proven to be fairly limited. Furthermore, the current era of genomics is characterized by organisms for which the entire set of pairwise genetic interactions are only partially known. Although high-throughput methods will soon be able to systematically cover all of the pairwise interactions in the *S. cerevisiae *genome, the research community is still faced with the task of mapping interactions in less tractable organisms and for interactions between three or more genes. Therefore, the goal of creating a genome-wide map of genetic interactions remains very important, as increasingly it appears that synthetic interactions amongst genes are representative of functional redundancy between the complexes and pathways to which the genes belong [11,12,14]. In summary, our method might be particularly useful in situations were the genetic interactome of an organism is only partially mapped, or for lesser-studied organisms for which homologous information from a close relative is available. The performance gains associated with the random walk method were sustained when using an alternate classifier. To demonstrate this feature, we use the logistic regression classifier [21], since it handles non-linear relationships between variables. Using the logistic regression classifier implementation from Weka [19], for *S. cerevisiae*, the random walk method obtained an AUC of 0.926 versus an AUC of 0.907 for the 2-hop method. For *C. elegans*, the random walk and 2-hop methods resulted in scores of 0.916 and 0.821, respectively. The logistic regression classifier produced lower AUC values for all data points as compared to the decision tree classifier. The higher scores obtained by the decision tree classifier might be attributable this classifier's inherent ability to capture second order interactions between variables. For example, we observed that the performance of the decision tree classifier peaked upon the incorporation of non-interaction scores, whereas the performance of the logistic regression classifier was less consistent in this regard (see tables 1 and 2 in additional file 1).

**Table 1:** Biological Datasets

Data Type	*S. cerevisiae*	*C. elegans*
GO	Yes	No
PPI	Yes	Yes
SSL	Yes	Yes
Yeast Orthologs	No	Yes
Human Orthologs	No	Yes
Co-expression	No	Yes

The performance of the Zhong *et al*. classifier was relatively weak compared to either of the two methods utilizing network topology. We suspect that the approach of Zhong *et al*., which uses Bayes' formula to derive a likelihood ratio to score gene pairs for each piece of evidence, would be better suited for predicting general functional relationships between genes. Indeed, Lee *et al*. [22] recently published work using a very similar framework to predict functional similarities between gene pairs. However, in contrast to Zhong *et al*., the authors used GO functionality as their training data, of which there is considerably more information that is better suited for measuring the degree to which proteins may function coherently. And we reiterate that, for the purpose of predicting genetic interactions, the random walk method offers the advantage of combining genetic interaction data with information regarding functional network topology.

While this study focuses on predicting genetic interactions, using random walks as a method for capturing properties of biological networks may be applied to other areas of bioinformatics. One potential application concerns the prediction of novel transcription factor-gene interactions, which was recently implemented using a 2-hop scheme [23]. Additionally, our findings will hopefully encourage the reporting of non-interactions for all studies in reverse and forward genetics. We found that non-interactions considerably improve the performance of our classifier, and these gains represent a lower bound on the potential benefit, as non-interaction data was not available for some of the studies.

## Conclusion

We presented a method based on applying random walks to biological networks to capture aspects of network topology that can be used to classify potential genetic interactions as either synthetic lethal or non-interacting. Our method, which is generalizable to all types of biological networks, is likely to perform well with limited information, as estimated by holding out large portions of the SSL interactions and non-interactions.

## Methods

### Biological data

We chose to test the performance of our method on two well-studied model organisms, as it allows testing for consistency across organisms and their respective biological networks. The *S. cerevisiae *dataset is composed of 3 networks from GO, PPI and SSL interaction data. Co-expression, PPI, SSL, human homologs and yeast homologs comprise the *C. elegans *study (Table 1).

#### Genetic interaction data

Data on synthetic sick or lethal (SSL) interactions were aggregated from several studies. For *S. cerevisiae*, we collected 12, 397 synthetic lethal interactions from the 2.0.31 version of the BioGRID database [24]. In addition to these interactions, we collected 9, 472 synthetic sick or lethal interactions from the Collins *et al*. [8] study. Note that this study provided a scoring matrix from which we counted scores that were < -3 as SSL. We also collected 97, 450 pairwise interactions that scored > 0, which we categorize as non-interactions. 563 SSL interactions and 17, 498 non-interactions were collected from a study conducted by Davierwala *et al*. [25]. Lastly, we obtained 611, 509 non-interactions from the Tong *et al*. [7] study, for which the SSL interactions are already included in the BioGRID database.

For *C. elegans*, we obtained 1, 246 SSL interactions and 3, 771 non-interactions from the Byrne *et al*. study [26], which used RNAi knockdown to test for synthetic sickness or lethality. Similarly, Lehner *et al*. [27] generated 338 SSL interactions and 57, 306 non-interactions, also via RNAi knockdown. Finally, we incorporated 2, 279 hand-curated genetic interactions from wormbase version WS190. In total, there were 3, 863 SSL interactions and 58, 579 non-interactions for *C. elegans*.

#### Protein-protein interaction data

For *S. cerevisiae*, we used the high-confidence protein-protein interaction dataset generated by Batada *et al*. [28], which includes 9, 857 interaction pairs with representation from 4, 008 different genes. The authors produced the dataset by taking the intersection of multiple high-throughput protein interaction experiments. Specifically, the authors required that protein-protein interactions be present in two distinct experiments measured using two different experimental techniques (e.g. yeast two-hybrid, tandem mass spectrometry). Consequently, at the expense of lower coverage, we reduce the potentially negative impact of false positive protein-protein interactions on our classification scheme.

For *C. elegans *protein-protein interactions, we used the worm interactome [29] dataset, which covers 1, 371 interactions between 1, 136 proteins.

#### Homologs of *C. elegans *proteins

For *C. elegans*, we incorporated homologs of worm genes that are known to interact in other organisms (also termed "interologs"). These datasets were procured by Marcotte and colleagues [22]. In total, there are 30, 098 interactions between 3, 145 genes of *H. sapiens *homologs and 56, 193 interactions between 2, 627 genes of *S. cerevisiae *homologs.

#### Co-expression network of *C. elegans *genes

For the co-expression network, we again included data procured by Marcotte and colleagues [22]. The co-expression network prepared in their study includes 287, 130 interactions for 14, 491 genes.

#### Gene Ontology data for *S. cerevisiae*

Gene Ontology data [30] for *S. cerevisiae *was obtained from the project website. In order to construct a gene network for the GO data, we flattened out the information into pairwise interactions using the "has a" relationship rule implemented by Marcotte and colleagues [31]. This produced a dataset of 66, 174 pairwise interactions with representation from 3, 515 genes.

### Implementation of existing methods

#### 2-hop method

We implemented the 2-hop characteristics from the Wong *et al*. method as described in the manuscript, which were subsequently incorporated into Weka's J48 decision tree classifier. We opted to exclude the functional information for two reasons: Figure 1 from Wong *et al*. indicates that the 2-hop characteristics provide nearly all of the predictive power, while the predictive ability of the functional information was very limited. Secondly, if desired, the random walk method can be complemented with other types of information, just as the 2-hop characteristics were in the Wong *et al*. study. In summary, both the random walk and 2-hop methods were applied to the same datasets, trained via 5-fold cross-validation with the aforementioned training sets, and scored with a decision tree classifier.

#### Zhong *et al*. method

We implemented the method of Zhong *et al*. by deriving likelihood scores from Bayes' formula, as described in the manuscript. There were, however, two significant differences. The first concerns the training data. Zhong *et al*. used a combination of 1, 816 genetic interactions and 2, 878 (Y2H) physical interactions as their positive training set. For the negative training set, the authors generated 3, 296 gene pairs that were linked in cis from genetic mapping experiments. In light of the two recent system-wide studies [26,27] for *C. elegans*, our implementation of the Zhong method uses the 3, 863 SSL interactions and 58, 579 non-interactions that were compiled for the random walk and 2-hop systems. Secondly, we did not use any of the ortholog data from either *S. cerevisiae *or *D. melanogaster*, due to the fact that none of the datasets exceeded a 2 percent threshold for coverage. We note that, in general, the coverage of any technique that relies solely on pairwise evidence and pairwise predictions will be quite limited. Still, we adjusted for the lower coverage associated with the Zhong *et al*. method by filtering out instances in the training sets that did not have any evidence. This reduced the dataset to 3, 332 SSL interactions and 33, 412 non-interactions. In contrast, both the random walk and 2-hop methods have representation for at least one dataset in each of the 3, 863 SSL interactions and 58, 579 non-interactions in *C. elegans*. After performing logistic regression, the weighted scoring function is expressed as:

$$\begin{array}{l} \ln\left(\frac{1}{1-p}\right)=0.67(anatomy\_score)+0.53(cell\_group\_score)+0.32(cell\_score)+0.25(go\_score)+\\ 0.31(microarray\_kimbig\_score)+0.36(microarray\_smd\_score)+0.75(phenotype\_score)-6.2. \end{array}$$

## Authors' contributions

This work is supported in part by National Science Foundation grant IIS-0612327. We thank the anonymous reviewers for their feedback.

## Supplementary Material

Additional file 1 **Supplemental data of AUC values.** This supplement includes two tables, one each for the worm and yeast data. Each table displays AUC values for the four levels of information used (80, 20, 10, 5). Futhermore, AUC values are provided for both the decision tree and logistic regression classifiers. Click here for file
