# Supplementary material for: Predicting genetic interactions with random walks on biological networks
Source: BMC Bioinformatics. 2009 Jan 12;10:17. doi: 10.1186/1471-2105-10-17 (PMC2653491; doi:10.1186/1471-2105-10-17)
Supplement: Additional file 1 — Supplemental data of AUC values. This supplement includes two tables, one each for the worm and yeast data. Each table displays AUC values for the four levels of information used (80, 20, 10, 5). Futhermore, AUC values are provided for both the decision tree and logistic regression classifiers. [file 1471-2105-10-17-S1.pdf]

Yeast	1		2		3		4		5	
% Instances Scored	RW (+,-)		RW (+)		Continuous 2-hop (+,-)		Continuous 2-hop (+)		Binary 2-hop (+)	
	J48	Log. Reg.	J48	Log. Reg.	J48	Logistic	J48	Logistic	J48	Logistic
80	0.969	0.926	0.952	0.947	0.891	0.893	0.874	0.907	0.788	0.908
20	0.949	0.920	0.832	0.940	0.862	0.868	0.834	0.869	0.829	0.867
10	0.907	0.918	0.799	0.933	0.829	0.848	0.800	0.833	0.795	0.831
5	0.841	0.913	0.743	0.922	0.781	0.816	0.781	0.753	0.750	0.779

Worm	1		2		3		4		5	
% Instances Scored	RW (+,-)		RW (+)		Continuous 2-hop (+,-)		Continuous 2-hop (+)		Binary 2-hop (+)	
	J48	Logistic	J48	Logistic	J48	Logistic	J48	Logistic	J48	Logistic
80	0.984	0.916	0.938	0.929	0.871	0.842	0.793	0.821	0.792	0.811
20	0.972	0.891	0.897	0.905	0.745	0.792	0.718	0.736	0.699	0.710
10	0.947	0.868	0.853	0.868	0.649	0.761	0.647	0.681	0.656	0.673
5	0.892	0.828	0.775	0.818	0.594	0.598	0.598	0.614	0.608	0.608

1 Supplementary Information

The two supplementary tables display AUC values for both classifiers at each data point for the yeast and worm datasets. Column 5 ("Binary 2-hop") is an implementation of the Wong et al. method, where values are represented as a binary 0 or 1. Column 4 (Continuous 2-hop) is a derivative of the Wong et al. method, where variables are represented as continuous variables. The continuous variable version of the 2-hop method performs at the same level or better than the binary version at nearly every data point. Column 3 (Continuous 2-hop with non-interactions included) demonstrates the performance gain associated with incorporating non-interactions. Column 2 represents the random walk method scored on SSL interactions only. Column 1 is the random walk as presented in the manuscript, where both SSL and non-interactions are utilized.

We chose to compare our method to the continuous 2-hop version (column 2) instead of the binary 2-hop version (column 1) for two reasons. First, comparison to the continuous 2-hop version offers a more direct comparison to our random walk method, controlling for the possibility that the use of continuous variables is responsible for the performance gains. Second, when applying the decision tree classifier to the binary 2-hop method while scoring with 80% of the genetic interactions, we needed to adjust the weighting of the class predictions from the typical 1:1 to 1:1.3 in favor of the SSL class. Without re-weighting the class prediction matrix, every leaf in the decision tree predicted the non-interacting class, producing an AUC of 0.5. This stems from the fairly common problem of instance imbalance in the training dataset; in the case of predicting genetic interactions, more than 90% of the instances belong to the non-interaction class. We note that this problem only arose in the case of the binary 2-hop method. This should not be unexpected since the variables are binary, and after scoring on a sufficient number of instances, the non-interacting scores begin to resemble the SSL scores.

Analyzing all of these methods simultaneously can provide insight into the marginal benefits of each aspect of our method. First, it is apparent that the continuous variable version of the 2-hop method offers slightly better performance in the case of the decision tree classifier, though the effects are especially small in the case of the logistic regression classifier. Second, moving from column 4 to column 3 (or from column 2 to column 1), incorporating non-interaction data benefits the decision-tree classifier but has a negligible or even detrimental effect on the logistic

regression classifier. This would tend to suggest that the decision tree classifier is better at detecting combinatorial relationships between variables. Lastly, moving from columns 3-5 to columns 1-2, it is clear that the random walk method of capturing network topology is responsible for the bulk of the performance gains. For the optimal decision tree classifier, the random walk method, while scoring only 10% of the instances, outperforms any of the 2-hop variants at 80% of the information.
